# Triple-Band Dual-Sense Circularly Polarized Hybrid Dielectric Resonator Antenna

**DOI:** 10.3390/s18113899

**Published:** 2018-11-12

**Authors:** Amir Altaf, Munkyo Seo

**Affiliations:** Department of Electrical and Computer Engineering, Sungkyunkwan University, Suwon 440-746, Korea; amiraltaf@dongguk.edu or amiraltaf@skku.edu

**Keywords:** dielectric resonator antenna (DRA), hybrid antenna, multi-band, square microstrip ring, triple-band circular polarization

## Abstract

In this paper, a triple-band dual-sense circularly polarized (CP) hybrid dielectric resonator antenna is proposed. A modified hexagonal dielectric resonator (DR) is top-loaded with a square microstrip ring (SMR). A vertical-tapered-strip connected to a 50-Ω microstrip line is used to excite the proposed antenna. It is found that the lower and central CP bands correspond to left-handed circular polarization and are produced by the TM${}_{11}$ and TE${}_{111}$ modes of the SMR and modified hexagonal DR, respectively. The upper CP band is formed by the combination of the quasi-TM${}_{21}$ mode of the SMR and quasi-TE${}_{111}$ mode of the DR that exhibits right-handed circular polarization. The measurement results of the fabricated prototype show triple-band response for |S${}_{11}|<$ −10 dB with impedance bandwidths (IBWs) of 17.4% (1.75–2.03 GHz), 28.13% (2.23–2.96 GHz), and 2.97% (3.65–3.76 GHz) in the lower, central, and upper bands, respectively. The measured 3 dB axial ratio bandwidths lying within −10 dB IBWs are 3.69% (1.86–1.93 GHz), 5.46% (2.67–2.82 GHz), and 2.15% (3.68–3.76 GHz) along with the peak gains of 5 dBic, 5.28 dBic, and 2.36 dBic in the lower, central, and upper bands, respectively.

## 1. Introduction

Dielectric resonator antennas (DRAs) possess attractive features like compact dimensions, high efficiency, negligible losses, relatively wide bandwidth, and design flexibility [1]. These advantages are further enhanced when DRAs with circularly polarized (CP) radiation are employed as they are insensitive to polarization mismatch and cancel the effect of multipath interference [2,3]. The antennas with dual-sense circular polarization at different bands are famous for applications like multimode GPS, satellite digital audio broadcast systems, and indoor wireless communication systems [4,5]. A recent study shows that the dual-sense CP antennas for GNSS application can mitigate short delay multipath effectively as compared with the same-sense CP antennas [6].

The CP DRAs are designed by excitation of a pair of orthogonal modes of same amplitude in the dielectric resonator (DR), which is achieved by either modifying the shape of the DR [7,8], or utilizing the appropriate feeding method. The feeding method can be of single-point or dual-point type where the former is simpler and more compact than the latter but the 3 dB axial ratio bandwidth (ARBW) is narrower [9,10,11]. To cope with the demands of modern communication systems, the single-point fed dual-band CP DRAs have been demonstrated in recent times. One such technique involves the separate excitation of fundamental and higher modes of the DR [12,13,14,15,16]. The other includes the use of hybrid configuration where modes of the DR and coupling slot are separately excited [17,18]. For instance, a dual-band CP hybrid antenna is proposed where the lower CP band is produced by the DRA and upper band by the cross-slot [17]. However in the aforementioned designs, the lower CP band is dependent on the TE${}_{111}$ mode of the DR, therefore, the size of the antenna would be increased to operate at lower frequencies for a given value of dielectric constant. Yong at el. designed a dual-band CP zonal-slot/DRA hybrid antenna where the lower CP comes from the zonal-slot and upper one from the DR [18], but the size was increased by the use of a rectangular cavity. Although several dual-/wide-band CP DRAs have been proposed, only one triple-band CP DRA with 3 dB ARBWs of 1.55%, 4.96%, and 0.8% is reported [19].

In this paper, a triple-band dual-sense circularly polarized hybrid DRA is proposed. A modified hexagonal DR is top-loaded by a square microstrip ring (SMR) and fed by a vertical-tapered-strip that is connected to a 50-Ω microstrip line. It is found that the lower CP band is produced by TM${}_{11}$ mode of the SMR, the central CP band by TE${}_{111}$ mode of the modified hexagonal DR, and the upper band by the combination of quasi-TM${}_{21}$ and quasi-TE${}_{111}$ modes of the SMR and modified hexagonal DR, respectively. The field distributions show that the lower and central CP bands exhibit left-handed circular polarization (LHCP) while right-handed circular polarization (RHCP) is obtained in the upper CP band. The measured −10 dB impedance bandwidths (IBWs) cover applications like PCS-1900 working at 1.85–1.99 GHz, DARS broadcasting at 2.31–2.36 GHz, ISM band with bandwidth of 2.4–2.483 GHz, airport surveillance radars working at 2.7–2.9 GHz, and some parts of the WiMAX medium band (3.2–3.8 GHz). Some portion of PCS-1900 (1.86–1.93 GHz), airport surveillance radars (2.7–2.82 GHz), and WiMAX (3.68–3.78 GHz) are covered by the CP bands. Nonetheless, the gains and axial ratios (ARs) across all bands can be enhanced by developing a sequentially rotated array of the proposed antenna. Details of the antenna design, parametric analysis, and measurement results are discussed in the subsequent sections.

## 2. Antenna Configuration

**Figure 1:**
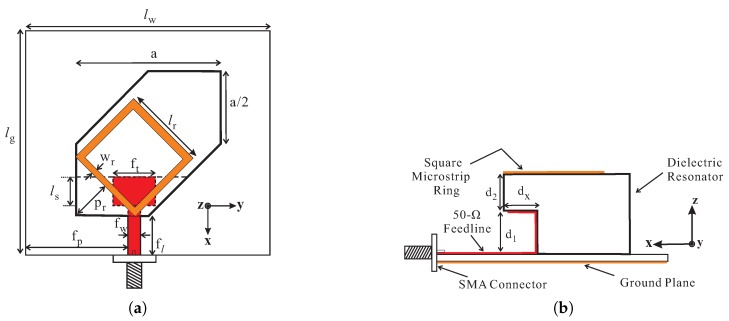
Geometry of the proposed hybrid dielectric resonator antenna (DRA): (**a**) Top view; (**b**) Side view. Dimensions in mm: (*a* = 35.4, $d_{1}$ = 13, $d_{2}$ = 11, $d_{x}$ = 9.5, $f_{l}$ = 17.3, $f_{p}$ = 35, $f_{t}$ = 10.5, $f_{w}$ = 3, $l_{g}$ = 70, $l_{r}$ = 20.5, $l_{s}$ = 8, $l_{w}$ = 80, $w_{r}$ = 1.5, $p_{r}$ = 10.3).

Figure 1 shows the geometry of the proposed antenna, which consists of a modified hexagonal DR that is top-loaded by an SMR, an RF-35 substrate with relative permittivity ($\varepsilon_{sub}$) of 3.5 and height ($h_{sub}$) of 1.52-mm, a ground plane, and 50-Ω microstrip line connected to a SMA connector from one end and vertical-tapered-strip from the other. The modified hexagonal DR is made up of Alumina having a relative permittivity ($\varepsilon_{dr}$) of 9.9, loss tangent of 0.0002, and is placed at the center of the top side of the substrate. Initially, a rectangular DR with dimensions of $a\times a\times (d_{1}+d_{2})$ is taken. To realize the geometry of the proposed modified hexagonal DR, the rectangular DR is truncated by a/2 from the opposite sides and further carved from the lower side by $d_{x}$ and $d_{1}$ along the *x*- and *z*-axes. An SMR with side length of $l_{r}$ and width of $w_{r}$ is placed at the top of the modified hexagonal DR such that the center is aligned with the diagonal of the modified hexagonal DR and the distance between the lower edge of the DR to the center of the nearest side of SMR is $p_{r}$. A 50-Ω microstrip feedline of width $f_{w}$ and length $f_{l}+d_{x}$ is printed on the upper side and is positioned at $f_{p}$ from the left bottom edge of the substrate. A vertical-tapered-strip having vertical-strip of length $d_{1}$, tapered-width of $f_{t}$, and upper *x*-directed length of $l_{s}$ is connected to 50-Ω line to excite the proposed hybrid DRA. The upper horizontal strip notably improves the matching at the lower and upper bands to fully incorporate the 3 dB ARBWs. The ground plane of dimensions $l_{w}\times l_{g}$ lies at the bottom side of the substrate. The optimized dimensions are mentioned in the caption of Figure 1.

**Figure 2:**
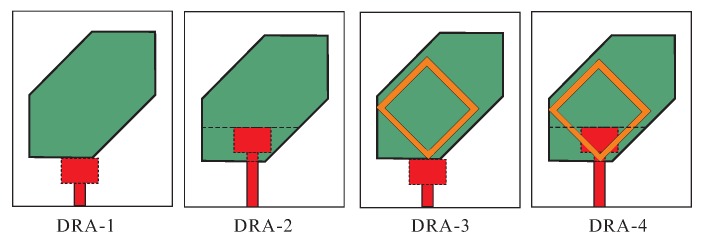
Configurations of DRA-1–DRA-4.

**Figure 3:**
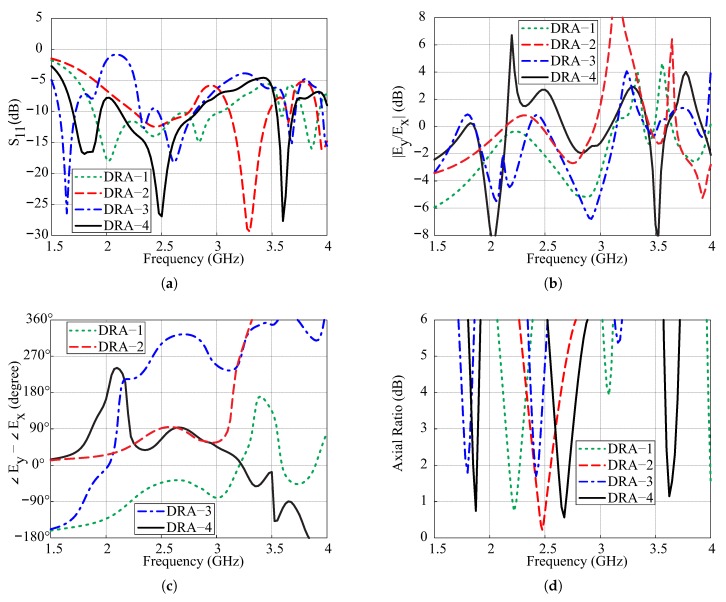
Comparison of the simulated results of DRA-1–DRA-4: (**a**) Reflection coefficients; (**b**) Amplitude ratios; (**c**) Phase differences; (**d**) Axial ratios.

Figure 2 depicts the geometries of DRA-1–DRA-4 for the explanation of the proposed geometry. All designs have the same length of the vertical-tapered-strip while DRA-1 and DRA-3 have a smaller 50-Ω feedline than the remaining two antennas. Figure 3 plots the comparison results regarding simulated −10 dB IBWs, amplitude ratios, phase differences (PDs), and ARs. From Figure 3a, DRA-1 has a wide −10 dB IBW around 2.4 GHz. By carving the DR and extending the length of the feedline to form DRA-2, a dual-band response is observed. DRA-3 is designed by placing the SMR on the top of DRA-1 and a new −10 dB impedance band (IB) around 1.65 GHz is generated by the SMR. The proposed design (DRA-4) is obtained by cutting the DRA-3 and increasing the length of the 50-Ω line. The simulated result from Figure 3a reports the triple-band response with −10 dB IBWs of 14.28% (1.69–1.95 GHz), 28.67% (2.12–2.83 GHz), and 4.42% (3.54–3.7 GHz) in the lower, central, and upper bands, respectively. Due to the hexagonal shape of all DRs, the two orthogonal modes of the same magnitude and quadrature PD are excited to produce the circular polarization. The graphs of amplitude ratio and PD versus frequency for all designs are plotted in Figure 3b,c. DRA-1 and DRA-2 fulfill the circular polarization condition only around 2.2 GHz and 2.45 GHz, respectively, to produce a single CP band. The DRA-2 produces a CP band at a little higher frequency from DRA-1 due to the lower value of effective dielectric constant. For DRA-3, the amplitude ratios are approximately 0.9 dB, and PDs are −88° and 268° at the frequencies of 1.8 GHz and 2.42 GHz, respectively, where the lower band is produced by the SMR and the upper one by the hexagonal DR. The CP bands of DRA-3 in the current configuration do not lie within −10 dB IBWs. However, the matching at the CP frequencies can be improved by optimizing the feeding structure. Nonetheless, the simulated results of DRA-3 are presented to clearly elaborate the reason for employing modified hexagonal DR in the proposed design. In the case of the proposed design (DRA-4), there are three frequencies (1.865 GHz, 2.675 GHz, and 3.625 GHz) where the amplitude ratios and PDs are simultaneously close to 0 dB and ±90°, respectively. Therefore, the proposed design is a triple-band CP antenna. It is noted in Figure 3c that the upper CP band of the proposed antenna is out of phase as compared to the remaining two bands suggesting that the sense of circular polarization is changed at the corresponding band. For ARs as in Figure 3d, the 3 dB ARBWs of the DRA-4 in the ascending order are 2.68% (1.84–1.89 GHz), 5.99% (2.59–2.75 GHz), and 2.47% (3.60–3.69 GHz). Furthermore, comparing DRA-4 with DRA-2, it is noted that the SMR loading slightly shifts the CP band of the DR towards the higher frequencies.

To find the resonance frequency of the TE${}_{111}$ mode, the modified hexagonal DR is approximated to a rectangular DR to find equivalent permittivity ($\varepsilon_{eq}$) [20]

(1) $$\varepsilon_{eq}=\frac{\varepsilon_{dr}\times V_{dr}+V_{air}}{V_{dr}+V_{air}},$$

where $\varepsilon_{dr}$ is the relative permittivity of the DR and where $V_{dr}$ and $V_{air}$ represent the volume of the DR and air, respectively. Taking into account the relative permittivity of the substrate, the resultant relative permittivity ($\varepsilon_{re}$) is given by [21]

(2) $$\varepsilon_{re}=\frac{d_{t}}{\frac{d_{1}+d_{2}}{\varepsilon_{eq}}+\frac{h_{sub}}{\varepsilon_{sub}}},$$

where $\varepsilon_{sub}$ and $h_{sub}$ represent the relative permittivity and height of the RF-35 substrate, respectively, and where $d_{t}$ denotes the net height that is given by

(3) $$d_{t}=\left(d_{1}+d_{2}+h_{sub}\right).$$

The *x*-directed wavenumber for TE${}_{111}$ mode of the DR can be found using (1)–(3) with the dielectric waveguide model [22] as follows:

(4) $$a\cdot tan\left(\frac{a\cdot k_{x}}{2}\right)=\sqrt{\left(\varepsilon_{re}-1\right)k_{0}^{2}-k_{x}^{2}},$$

where

(5) $$k_{0}^{2}\cdot \varepsilon_{re}=k_{x}^{2}+k_{y}^{2}+k_{z}^{2},\;k_{y}=\frac{\pi }{a},\;and\;k_{z}=\frac{\pi }{2\cdot d},$$

where *a* is the length of the modified hexagonal DR and where $k_{x}$, $k_{y}$, and $k_{z}$ represent the wavenumbers along the *x*-, *y*-, and *z*-axes. The calculated value of $k_{x}$ = 0.0583 is obtained using the MATLAB code available in [23]. The resonance frequency($f_{dr}$) of the DR is calculated by

(6) $$f_{dr}=\frac{c\cdot k_{0}}{2\pi }.$$

The calculated resonance frequency for the TE${}_{111}$ mode of the DR without SMR is 2.45 GHz as compared to the simulated center value of 2.49 GHz in the central band. The resonance frequency ($f_{r}$) for TM${}_{11}$ mode of the SMR is given as follows [24]:

(7) $$f_{r}=\frac{c}{4\left(l_{r}-w_{r}\right)\sqrt{\varepsilon_{eff}}},$$

where $l_{r}$ and $w_{r}$ represents the length and width of the SMR, respectively, and $\varepsilon_{eff}$ is the effective permittivity that is calculated by

(8) $$\varepsilon_{eff}=\varepsilon_{rc}-\frac{\varepsilon_{rc}-\varepsilon_{e}\left(0\right)}{1+{\left(f/f_{p}\right)}^{m}},$$

where details of the parameters in (8) can be found in [25].

Using the above equations, the calculated value of $f_{r}$ is 1.83 GHz as compared to the simulated center frequency ($f_{sim}$) of 1.82 GHz for the lower −10 dB IB. The simulated center frequency of TM${}_{21}$ mode lies at 3.62 GHz, which is ≈ 2$\times f_{sim}$.

**Figure 4:**
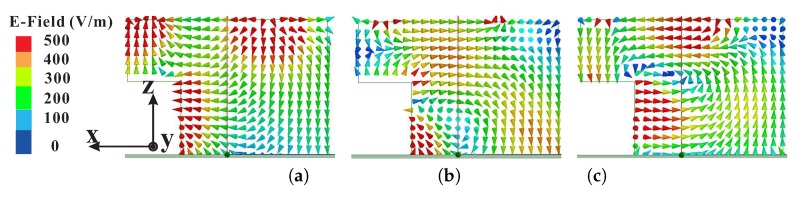
Simulated electric field (E-field) distribution at the center of the modified hexagonal dielectric resonator (DR) for axial ratios (ARs) minima: (**a**) 1.865 GHz; (**b**) TE${}_{111}$ mode at 2.67 GHz; (**c**) quasi-TE${}_{111}$ mode at 3.645 GHz.

**Figure 5:**
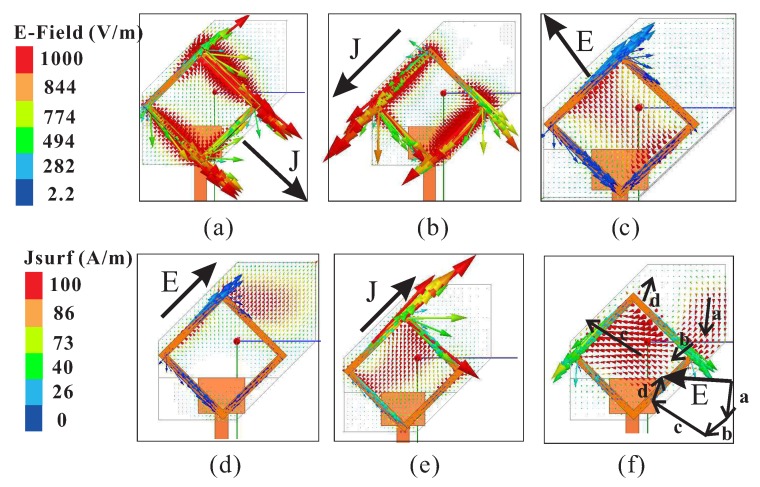
Simulated E-field $\vec{E}$ and surface current $\vec{J}$ distributions with respect to phase angle in the proposed design: (**a**) 1.865 GHz ∠0°; (**b**) 1.865 GHz ∠90°; (**c**) 2.67 GHz ∠0°; (**d**) 2.67 GHz ∠90°; (**e**) 3.645 GHz ∠0°; (**f**) 3.645 GHz ∠90°.

Figure 4 presents the simulated electric field (E-field) distributions at the center of the modified hexagonal DR for the center frequencies of ARs minima as seen from the *y*-axis. It is observed that the E-field distribution at 1.865 GHz does not resemble the TE${}_{111}$ mode of the rectangular DR signifying that the lower CP band is produced by the SMR. For the central and upper CP bands, the E-field resembles the TE${}_{111}$ and quasi-TE${}_{111}$ modes of the DR, respectively. To determine the sense of circular polarization, the simulated E-field distributions on the top surface of the DR ($\vec{E}$) and surface current distribution of the SMR ($\vec{J}$) versus the phase angle are plotted in Figure 5. At 1.865 GHz, the dominant contribution is from vector $\vec{J}$ with a clockwise sense of rotation as the phase angle changes from ∠0° to ∠90°, thus producing LHCP (see Figure 5a,b). Similarly, the vector $\vec{E}$ at 2.67 GHz rotates in a clockwise direction with the increase in the phase angle to produce LHCP as shown in Figure 5c,d. At 3.645 GHz ∠0°, the surface current of the SMR is stronger due to the radiation from asymmetric TM${}_{21}$ (quasi-TM${}_{21}$) mode; hence, the resultant field is along vector $\vec{J}$ as shown in Figure 5e. At 3.645 GHz ∠90°, the radiation from the DR dominates the fields of the SMR. The direction of the resultant vector $\vec{E}$ is obtained by adding the major field distributions ($\vec{a}$,$\vec{b}$, $\vec{c}$, $\vec{d}$) on the top surface of the DRA. The resultant vector $\vec{E}$ is rotated in an anticlockwise direction as compared to vector $\vec{J}$ for 3.65 GHz ∠0° to produce RHCP radiation. It is worth mentioning that the sense of circular polarization is controlled by the direction of microtrip feeding and dimensions of the ground plane. In order to reverse the sense of circular polarization, the modified hexagonal DR should be truncated by $d_{x}$ along the *y*-axis. Also, the feedline will be aligned with the *y*-axis and the values of $l_{g}$ and $l_{w}$ will be interchanged.

## 3. Parametric Analysis

The parametric analysis of the proposed design is performed using the ANSYS High Frequency Structure Simulator (HFSS) software. The parameters of interest are the position $p_{r}$, length $l_{r}$, and width $w_{r}$ of the SMR, and their effects on the simulated reflection coefficients and ARs are analyzed. During this analysis, only the parameters of interest are varied.

**Figure 6:**
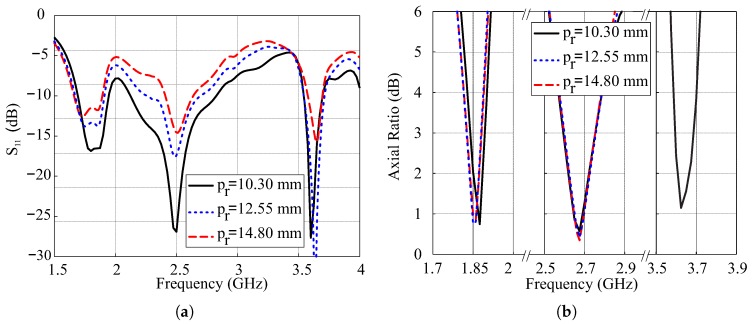
Simulated effects of variations in position $p_{r}$ of the square microstrip ring (SMR): (**a**) Reflection coefficients; (**b**) Axial ratios.

Figure 6 shows the effects of varying the position $p_{r}$ of the SMR with respect to the lower edge of the modified hexagonal DR. With the increase in value of $p_{r}$, the SMR moves towards the center of the DR. It can be seen that −10 dB IBW of the central band is particularly affected by this variation and the bandwidth continues to decrease with the increase in $p_{r}$ as shown in Figure 6a. It is evident from Figure 6b that the upper CP band is very sensitive to the position of SMR while the remaining two remains almost unchanged. The triple-band CP is obtained only for $p_{r}$ = 10.30 mm, while dual-band CP response is obtained for other values of $p_{r}$.

**Figure 7:**
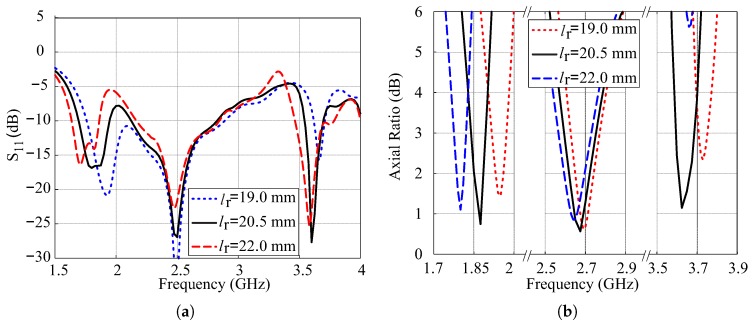
Simulated effects of variations in length $l_{r}$ of the SMR: (**a**) Reflection coefficients; (**b**) Axial ratios.

The effects of variations in length $l_{r}$ of the SMR on the simulated reflection coefficients and ARs are depicted in Figure 7. The −10 dB IBs of the TM${}_{11}$ and quasi-TM${}_{21}$ modes move following the variations in the $l_{r}$, while the resonance band of the modified hexagonal DR remains almost constant. For $l_{r}$ = 19 mm, the lower and central IBs combine to produce wide-band performance. In case of ARs as depicted in Figure 7b, the CP band of TM${}_{11}$ mode of the SMR is affected the most by the variation in $l_{r}$. By increasing the value of $l_{r}$, the position of the central CP band slightly moves towards the lower frequencies due to the change of position of broadside null as shown in Figure 3. The upper CP band is very sensitive to this variation and dual-band CP is obtained as the value of $l_{r}$ exceeds 20.5 mm.

**Figure 8:**
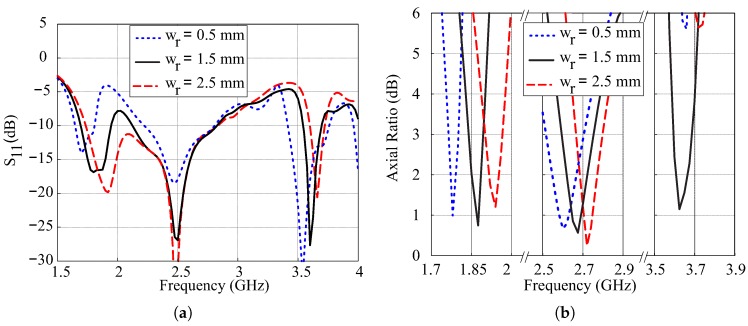
Simulated effects of variations in length $w_{r}$ of the SMR: (**a**) Reflection coefficients; (**b**) Axial ratios.

The simulated effects of variations in width $w_{r}$ of the SMR on reflection coefficients and ARs are plotted in Figure 8. From Figure 8a, it is observed that the lower and upper −10 dB IBs move towards lower frequencies with the decrease in the value of $w_{r}$. This is due to the confinement of the path of surface currents of the SMR resulting in lower resonance frequency and is also consistent with (7). It is also observed that wide-band characteristics are obtained for $w_{r}$ = 0.5 mm due to the combining of lower and central −10 dB IBs. In the case of ARs, the position of the lower and central CP bands varies in accordance with variations in $w_{r}$, and triple-band CP is obtained only for $w_{r}$ = 1.5 mm.

Based on the parametric study, the optimized values of $p_{r}$, $l_{r}$, and $w_{r}$ are 10.30 mm, 20.5 mm, and 1.5 mm, respectively.

## 4. Measurement Results and Discussion

**Figure 9:**
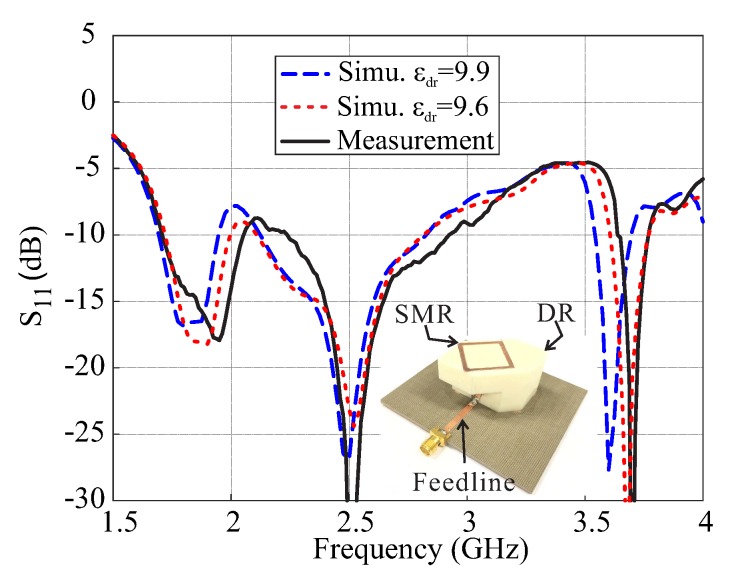
Simulated and measured reflection coefficients of the proposed antenna.

**Figure 10:**
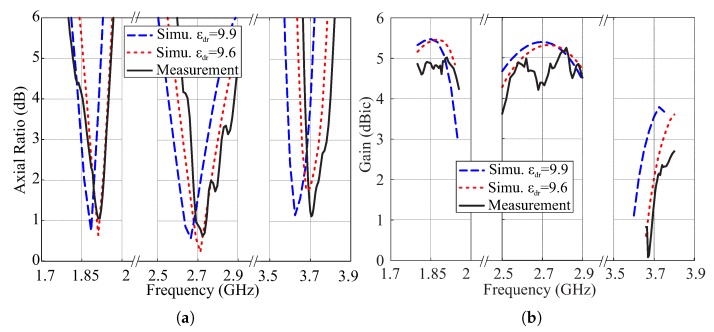
Simulated and measured results of the proposed antenna for θ=0°: (**a**) Axial ratios; (**b**) Gains.

A prototype of the proposed design is fabricated to measure the actual performance capabilities. The SMR and vertical-tapered-strip were realized by using the adhesive copper tape. The reflection coefficients are measured using the Agilent 8507C network analyzer, and comparative simulation and measurement results of the reflection coefficients and gains are presented in Figure 9. The measured −10 dB IBWs are 17.4% (1.705–2.03 GHz), 28.13% (2.23–2.96 GHz), and 2.97% (3.65–3.76 GHz) in the lower, central, and upper bands, respectively. These bands cover applications like PCS-1900 working at 1.85–1.99 GHz, DARS broadcasting at 2.31–2.36 GHz, the ISM band with bandwidth of 2.4–2.483 GHz, airport surveillance radars working at 2.7–2.9 GHz, and some parts of the WiMAX medium band (3.2–3.8 GHz). It is worth mentioning that the antenna can also be scaled and optimized for other frequency bands like [26]. The comparison of simulated and measured ARs for θ=0° are plotted in Figure 10a. The measured 3 dB ARBWs are 3.69% (1.86–1.93 GHz), 5.46% (2.67–2.82 GHz), and 2.68% (3.68–3.78 GHz) in the lower, central, and upper bands, respectively. The first two CP bands are entirely covered within their own −10 dB IBs while only 2.15% (3.68–3.76 GHz) is included for the upper band. The measured peak gains in the ascending orders for broadside direction are found to be 5 dBic, 5.28 dBic, and 2.36 dBic within the CP bands as shown in Figure 10b. As explained in Section 2, the $\varepsilon_{dr}$ of alumina is taken as 9.9 in the simulation while 99.7% alumina is used for the fabrication that has a dielectric constant of 9.6 as seen from the datasheet [27]. Therefore, the proposed design was resimulated assuming $\varepsilon_{dr}$ = 9.6 and results are also plotted in Figure 9 and Figure 10. The measurement results match the simulated results of $\varepsilon_{dr}$ = 9.6 reasonably well.

**Figure 11:**
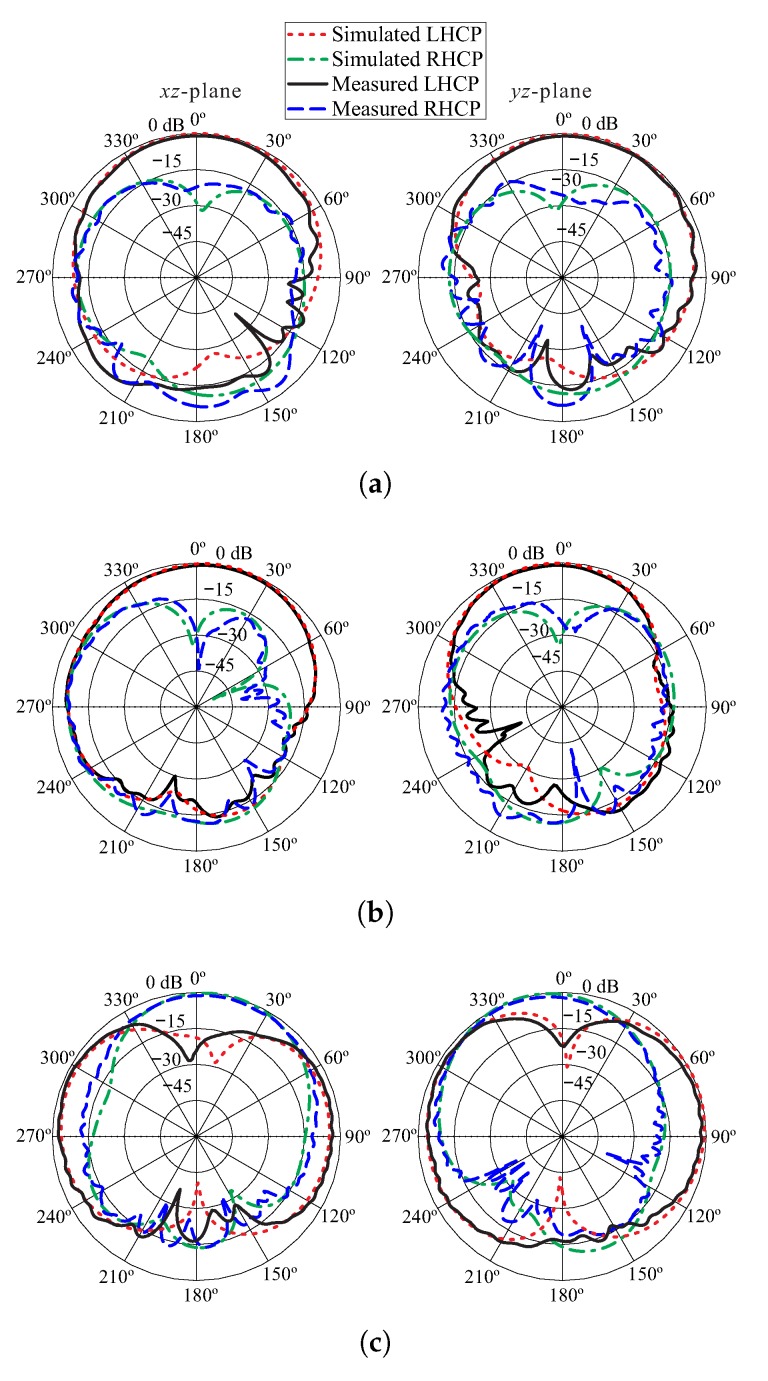
Normalized simulated and measured radiation patterns of the proposed antenna: (**a**) 1.9 GHz; (**b**) 2.73 GHz; (**c**) 3.7 GHz. Left-handed circular polarization (LHCP), right-handed circular polarization (RHCP).

The normalized simulated ($\varepsilon_{dr}$ = 9.6) and measured radiation patterns of the proposed antenna at the measured AR minima (1.9 GHz, 2.73 GHz, and 3.7 GHz) for both xz- and yz-planes are depicted in Figure 11. The measurement for each band is performed separately considering only one cutting plane at a time. Therefore, the measured cross-polarization (*X*-pol) at both planes for θ = 0° is slightly different as compared to the simulated values due to the misalignment between the transmitter and antenna under test. Nonetheless, the measured co-polarization (co-pol) radiation is stronger by more than 20 dB from the *X*-pol in both planes for θ = 0°. The measured *X*-pol is better than those in [5].

**Figure 12:**
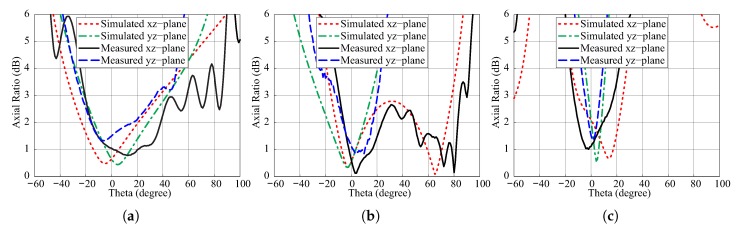
Simulated and measured axial ratios (ARs) versus angle theta of the proposed antenna: (**a**) 1.9 GHz; (**b**) 2.73 GHz; (**c**) 3.7 GHz.

**Figure 13:**
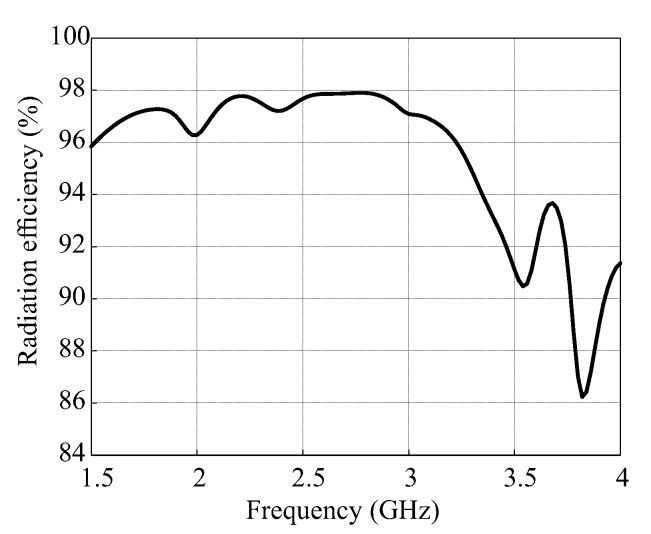
Simulated radiation efficiency of the proposed antenna.

Figure 12 describes the comparison of simulated and measured ARs concerning the observation angle theta at the frequencies of 1.9 GHz, 2.73 GHz, and 3.71 GHz. In ascending order of frequencies, the simulated 3 dB beamwidths for the xz-plane are found as 67.8°, 94.2°, and 35.2° and for the yz-plane are noted as 64.2°, 41.1°, 11.8°. In comparison, the measured values are 80°, 93.5°, and 29.2° for the xz-plane and 58°, 32.5°, and 12° for the yz-plane. The difference between the simulation and measurement results is caused by misalignment between the transmitter and antenna under test. Figure 13 plots the simulated efficiency versus frequency of the proposed antenna for $\varepsilon_{dr}$ = 9.6. The result shows that the radiation efficiency is greater than 90% for all bands.

Finally, a performance comparison of the proposed DRA with earlier works [12,13,14,15,17,18,19] is presented in Table 1. These structures are compared in terms of −10 dB IBWs, 3 dB ARBWs, and size. It should be noted that the $\lambda_{0}$ represents the wavelength with respect to the center frequency of the lower CP band. The earlier works [12,13,14,15,17,18] are some of the examples of dual-band CP DRAs. It is concluded from the comparison that the proposed DRA has a wider 3 dB ARBW at the second band from [12], wider −10 dB IBWs and 3 dB ARBWs from [13,15,17,18] along with the compact height from [13,18]. Compared with [14], the proposed work has higher gains and compact size. A triple-band omni-directional CP DRA with compact dimensions was presented in [19]. In comparison, the proposed DRA has wider 3 dB ABBWs and higher gains across all three bands.

## 5. Conclusions

In this paper, the design procedure of a triple-band CP hybrid DRA is presented and discussed. It is established that the lower CP band comes from the TM${}_{11}$ mode of the SMR, the central band from the TE${}_{111}$ mode of the DR, and the upper band from the combination of quasi-TM${}_{21}$ and quasi-TE${}_{111}$ modes of the SMR and DR, respectively. The experimental results yielded 3 dB ARBWs lying within −10 dB IBWs of 3.69% (1.86–1.93 GHz), 5.46% (2.67–2.82 GHz), and 2.15% (3.68–3.76 GHz) in the lower, central, and upper bands, respectively. The measured purity of co-pol radiation is more than 20 dB from the X-pol in the broadside direction at the measured frequencies. The proposed design due to triple-band CP characteristics can be adopted for applications like PCS-1900, airport surveillance radars, and WiMAX.

## Figures and Tables

**Table 1:** Comparison of the proposed DRA with earlier works. Impedance bandwidths (IBWs), axial ratio bandwidths (ARBWs).

Ref. No.	−10 dB IBWs (GHz) [%]			3 dB ARBWs (GHz) [%]			Gains (dBic)			Size ($\lambda_{0}$)
[12]	1.45–1.87 [25.3]	2.1–3 [35.3]	–	1.53–1.63 [6.3]	2.4–2.49 [3.68]	–	6.09	8.49	–	0.2 × 0.2 × 0.21
[13]	1.217–1.364 [11.4]	1.505–1.637 [8.4]	–	1.255–1.282 [2.1]	1.538–1.572 [2.2]	–	5.5	4.5	–	0.42 × 0.42 × 0.23
[14]	3.29–3.92 [17.47]	4.52–6.05 [28.94]	–	3.29–3.76 [13.33]	4.5–4.92 [7.81]	–	3.3	4.2	–	0.94 × 0.94 × 0.13
[15]	3.4–3.58 [5.16]	5.1–5.9 [14.55]	–	3.46–3.54 [2.28]	5.18–5.34 [3.04]	–	≈ 5.1	≈ 5.15	–	0.47 × 0.47 × 0.11
[17]	1.8–2.07 [14]	2.57–2.92 [12.8]	–	1.91–2.03 [3]	2.57–2.92 [3.5]	–	4.7	5.6	–	0.49 × 0.49 × 0.1
[18]	2.34–2.53 [7.8]	4.46–5.34 [17.96]	–	2.39–2.47 [3.29]	4.97–5.15 [3.56]	–	5.8	4.29	–	0.27 × 0.27 × 0.25
[19]	1.92–1.955 [1.55]	2.315–2.50 [7.68]	3.415–3.55 [3.87]	1.925–1.955 [1.55]	2.36–2.48 [4.96]	3.502–3.53 [0.8]	1.2	1.6	-1.5	0.32 × 0.32 × 0.15
Present work	1.705–2.03 [17.4]	2.23–2.96 [28.13]	3.65–3.76 [2.97]	1.86–1.93 [3.69]	2.67–2.82 [5.46]	3.68–3.76 [2.15]	5	5.28	2.36	0.44 × 0.5 × 0.16
